# Computational-experimental repurposing reveals synergistic sorafenib/hydroxychloroquine response in KRAS-mutant breast cancer

**DOI:** 10.1186/s40360-026-01122-2

**Published:** 2026-04-02

**Authors:** Mohammad M. Abdelwahab, Mahmoud Soliman, Amr Nassrallah

**Affiliations:** 1 Biotechnology Department, Faculty of Basic and Applied Sciences, Egypt-Japan University of Science and Technology (E-JUST), Alexandria, Egypt; 2 Biotechnology Department, Institute of Graduate Studies and Research, Alexandria University, Alexandria, Egypt; 3 Department of Pharmaceutics and Industrial Pharmacy, Faculty of Pharmacy, Ain Shams University, Cairo, Egypt; 4 PharmD Program, Faculty of Pharmacy, Egypt-Japan University of Science and Technology (EJUST), Alexandria, Egypt; 5 Biochemistry Department, Faculty of Agriculture, Cairo University, Giza, Cairo, 12613 Egypt

**Keywords:** KRAS, Autophagy, Kinases, Breast cancer, Sorafenib, Hydroxychloroquine

## Abstract

**Background:**

KRAS mutations are approximately 25% of human cancers, with particularly high incidence in breast and lung cancers, by constitutively triggering MAPK/ERK and PI3K/AKT pathways that advance proliferation, survival, and autophagy-mediated resistance. Targeting these pathways with kinase inhibitors upregulates autophagy, thereby diminishing therapeutic efficacy. Thus, combining kinase inhibitors with autophagy inhibitors offers a rational strategy to enhance antitumor benefits in KRAS-mutant malignancies. FDA-approved Sorafenib (multi-kinase inhibitor targeting Raf) and Hydroxychloroquine (autophagy inhibitor) show promise for repurposing, as Sorafenib induces autophagy leading to resistance, warranting combination testing in KRAS-mutant models. This study integrates computational modeling and in vitro assays to evaluate their synergistic potential in MDA-MB-231 breast and A549 lung cancer cells.

**Methods:**

Raf-Sorafenib stability was evaluated using RMSD, RMSF, Rg, hydrogen bonds, contact frequency, and MM/GBSA for binding free energy in molecular dynamics simulations (100 ns, GROMACS with CHARMM36 force field). MTT assays were used for cytotoxicity on MDA-MB-231, A549, and normal gingival fibroblasts (48 h treatment); Chou-Talalay Combination Index and Dose Reduction Index were used for synergy; Annexin V/PI flow cytometry was performed for apoptosis; PI staining was used for cell cycle; and ANOVA/Tukey’s test (GraphPad Prism, *p* < 0.05) was conducted for statistics.

**Results:**

Sorafenib bound Raf stably (RMSD ~ 0.25 nm protein/0.15 nm ligand, G_bind − 49.90 ± 2.69 kcal/mol), with persistent interactions (3–4 H-bonds, key residues VAL471, LEU513). IC50 values: Sorafenib 9.4 µM (MDA-MB-231), 12 µM (A549), 23.1 µM (fibroblasts); HQ 23.6/62.4/86.2 µM; SN:2HQ ratio showed synergy in MDA-MB-231 (CI = 0.32, DRI 9.36 Sorafenib/4.68 HQ at Fa = 0.5) but antagonism in A549 (CI > 1). Combination enhanced late apoptosis/necrosis (49.41%) in MDA-MB-231 with minimal normal cell cycle disruption.

**Conclusions:**

Sorafenib-HQ combination offers potent, context-specific synergy for KRAS-mutant breast cancer via Raf inhibition and autophagy blockade, enabling dose reductions and apoptosis enhancement. Tumor-type dependence (synergy vs. antagonism) highlights need for patient stratification; findings support repurposing with limited normal cell impact.

## Introduction

Wild cell proliferation, invasion, and metastasis are characteristics of cancer, a complex and all-encompassing disease [1]. ​​. With diverse alterations that contribute to oncogenesis, mutations in Kirsten rat sarcoma (KRAS) gene are particularly established and challenging to cure. KRAS mutations are found in approximately 25% of cancers among human, with high frequencies in breast, pancreatic, lung, and colorectal cancers [2]​. These mutations cause constitutive stimulation of the KRAS protein, which triggers downstream signaling pathways for instance the phosphatidylinositol 3-kinase (PI3K)/protein kinase B (AKT) and mitogen-activated protein kinase (MAPK)/extracellular signal-regulated kinase (ERK) pathways. These pathways promote cell survival, proliferation, and resistance to apoptosis, as well as aggressive tumor behavior and poor prognosis [3, 4]. In addition to promoting cell survival through these signaling pathways, KRAS mutations have been linked to autophagy, a cellular course that involves the mortification and recycling of ravaged organelles and proteins. Autophagy pieces a double function in cancer, as a cancer suppressor and as a mechanism that supports tumor cell survival under stress environment, for example nutrient deprivation or during chemotherapy [4, 5]​. The relationship between KRAS mutations and autophagy is multifaceted, with some studies signifying that KRAS-driven cancers may rely on autophagy for growth and survival, while others indicate that autophagy is expendable for the development of KRAS mutant cancers​ [3, 4]​.

Recent investigates have explored the curative capability of targeting autophagy in combination with kinase inhibitors to improve the effectiveness of tumor treatments​ [6, 7]​. Kinase inhibitors, such as those targeting the MAPK/ERK pathway, have shown promise in treating KRAS-mutated cancers but often face limitations due to compensatory signaling mechanisms and the development of resistance.

Autophagy inhibitors, like hydroxychloroquine (HQ), have been used to block autophagy, potentially sensitizing cancer cells to therapeutic agents by disrupting their ability to survive under stress conditions​ [3, 4]​. Combining kinase inhibitors with autophagy inhibitors has been shown to have a synergistic effect in a number of tumors, comprising MDA-MB-231 breast tumor and A549 lung tumor cells, indicating a possible method to overcome resistance and improve treatment outcomes​ [7, 8]​.

Sorafenib, a multi-kinase inhibitor, is an FDA-permitted drug for the cure of hepatocellular carcinoma and renal cell carcinoma, that exerts its mechanistic effect on Raf primarily by constraining its kinase action within the MAPK/ERK signaling pathway, while hydroxychloroquine (HQ) is FDA-permitted for the cure of malaria and autoimmune diseases​ [9–11]​. Studies have established that sorafenib induces autophagy, and the collaboration relating the drug and autophagy is accountable for the advancement of resistance​ [12].

Given their established clinical safety profiles, there is increasing interest in repurposing these agents for new oncological indications. This study marks the possibility of repurposing Sorafenib and HQ, two FDA-approved medications, as a combination therapy for malignancies with KRAS mutations. In particular, we assess their effects on normal cells as well as their individual and combined cytotoxic effects in the MDA-MB-231 breast and A549 lung tumor cell lines. By exploring the combined inhibition of kinase signaling and autophagy, this research seeks to provide a rationale for novel therapeutic strategies that leverage existing FDA-approved drugs to improve outcomes in aggressive, treatment-resistant cancers.

## Materials and methods

### Molecular dynamics simulation and MM/PBSA investigation

Following established computational procedures, the molecular dynamics (MD) simulation protocol and the ensuing binding free energy investigation were carried out. The order setup was conducted with the CHARMM-GUI solution builder​​ [13–15] employing the CHARMM36 force field. The Raf protein’s architecture was retrieved using PDB ID: 1UWJ. The ligand structure (Sorafenib) was submitted to the CGenFF server (https://cgenff.umaryland.edu/) using its canonical SMILES string, the generated topology had penalty scores < 10 for all parameters, indicating high-quality force field matching, Charges were assigned using the default CGenFF charge model with no manual modifications [16]. The initial protein-ligand complex was solvated in a TIP3P water model within a periodic boundary conditions (PBC) cubic box. The box dimensions were set to 64 × 64 × 64 Å, chosen to ensure a minimum distance of 10 Å between the protein and the box edges in all directions. The protein dimensions were approximately 45 × 40 × 40 Å, and this 64 Å box size satisfies this criterion. Na⁺ and Cl⁻ ions were added to achieve a physiological concentration of 0.15 M and system neutrality. Steric conflicts were eliminated using the steepest descent algorithm (5000 steps) for energy minimization. Positional restraints (400 kJ mol⁻¹ nm⁻² on the backbone and 40 kJ mol⁻¹ nm⁻² on the side chains) were applied only during the equilibration phases—consisting of 125 ps in the NVT ensemble followed by 125 ps in the NPT ensemble at 300.15 K—to gradually relax the system. These restraints represent standard methodological practice, allowing slow equilibration of solvent and ions while preventing unphysical movements of the protein backbone and side chains, with no biological relevance. No restraints were applied during the subsequent 100 ns production MD simulation, which was conducted in the NPT ensemble at 1 bar and 300.15 K using the Nosé–Hoover thermostat and Parrinello–Rahman barostat. During the MD simulation, all bonds involving hydrogen atoms were constrained using the LINCS algorithm. Long-range electrostatic interactions were treated with the particle mesh Ewald (PME) method, and a cutoff of 12 Å was applied for non-bonded interactions. Trajectory frames were saved every 2 ps for subsequent analysis. For hydrogen bond analysis, the GROMACS gmx hbond tool was used with a donor–acceptor distance cutoff of 3.5 Å and an acceptor–donor–hydrogen angle cutoff of 30° [17]. GROMACS tools were used to conduct trajectory analysis [18–20]. After alignment to the initial energy-minimized structure, root mean square deviation (RMSD) values for the protein backbone, ligand, and complete complex were calculated using gmx rms to assess conformational stability throughout the 100 ns production simulation. Root mean square fluctuation (RMSF) for Cα atoms was computed using gmx rmsf on the equilibrated trajectory segment was 100 ns to evaluate local flexibility. Note that the total production simulation length was 100 ns. Gmx gyrate was used to calculate the radius of gyration (Rg). Gmx h-bond was used to investigate intermolecular hydrogen bonding. Contact frequency (CF) analysis and trajectory visualization were performed with VMD and performed on the 100 ns trajectory [17]​, and protein–ligand interaction networks were mapped using the Prolif tool [21].

The gmx_MMPBSA module of the Molecular Mechanics Poisson–Boltzmann Surface Area (MM/PBSA) system was operated to predict the binding free energy (ΔG_bind) of Raf–Sorafenib complex [22–25]. The final 50 ns of the equilibrated trajectory yielded a total of 100 equally spaced snapshots. Solvent-accessible surface area (SASA) calculations were performed using the Generalized Born (GB) implicit solvent model (igb = 2) and the LCPO method. The binding free energy was calculated as follows:

$${\Delta}\mathrm{G}\mathrm{b}\mathrm{i}\mathrm{n}\mathrm{d}\text{}=\mathrm{G}\mathrm{c}\mathrm{o}\mathrm{m}\mathrm{p}\mathrm{l}\mathrm{e}\mathrm{x}\text{}-(\mathrm{G}\mathrm{p}\mathrm{r}\mathrm{o}\mathrm{t}\mathrm{e}\mathrm{i}\mathrm{n}\text{}+\mathrm{G}\mathrm{l}\mathrm{i}\mathrm{g}\mathrm{a}\mathrm{n}\mathrm{d}\text{})$$

where roles from van der Waals, electrostatic, polar solution, and non-polar soluation energies are included in each term. Energy measurements from 100 frames were used to calculate the standard error mean (SEM).

### Cell viability assay

Normal gingival fibroblast cells, lung A549 tumor cells, and breast MDA-MB-231 tumor cells were plated in 96-well plates at a density of 7000 cells per well in 100 µl of DMEM high glucose media (4.5 g/L) with 10% FBS and 1% penicillin/streptomycin at 5% CO2 and 37 °C. Following serum starvation, cells were treated for 48 h with sorafenib and hydroxychloroquine at various doses and combination ratios. MTT (5 mg/mL in 1×PBS) was inserted to the cells and harvested for three hours in 5% CO2 at 37 °C for cytotoxicity test via 3-(4,5-Dimethyl-2-thiazolyl)-2,5-diphenyl-2 H-tetrazolium bromide (MTT). To dissolve the formed formazan crystals, a 100 µL volume of DMSO was included to the cells and mixed. Eventually, the cell viability % was compared to the basic control and the absorbance was computed at 490 nm [26].​.

### Documentation of synergism, antagonism, and dose reduction in drug combination

The dose-response information for individual medications in MDA-MB-231 breast tumors and A549 lung tumor cells were used to determine the drug doses used in combination tests. As demonstrated by Chou, fraction affected (Fa) quantities were estimated as cell viability percentage inhibition in comparison to the control [27].

The Chou Tala lay Combination Index (CI) approach was used to identify if drug combinations were antagonistic, additive, or synergistic [27] The CompuSyn software (http://www.combosyn.com), which is rely on the next equation:

$$\mathrm{C}\mathrm{I}={\left.\left(\frac{\left(\mathrm{D}\right)1}{\left(\mathrm{D}\mathrm{x}\right)1}\right.\right)}^{}+\left.\left(\frac{\left(\mathrm{D}\right)2}{\left(\mathrm{D}\mathrm{x}\right)2}\right.\right)$$

where the concentrations of Drugs 1 and 2 in the combination to generate a Fa value of x are denoted by (D)1 and (D)2. When used as separate medicines, (Dx)1 and (Dx)2 represent the concentrations of Drug 1 and Drug 2 that generate the same effect (x). CI values reveal antagonism, additivity, and synergism, respectively.

The possibility for lowering the dosage of individual medications when taken together to attain a particular effect level in comparison to their use as single agents was measured using the Dose Reduction Index (DRI) [28]. In order to measure the likelihood that the doses of each medicine in a synergistic combination might be lowered through specific consequence level in comparison to the doses of each drug solely, the dose reduction index (DRI) was formally created. Using CompuSyn software (http://www.combosyn.com), the dose-reduction index (DRI) is primarily rely on the next equation:  

$$\eqalign{ & {\rm{CI}} = {\left. {\left( {{{\left( {\rm{D}} \right)1} \over {({\rm{Dx}}1}}} \right.} \right)^{}} + \left. {\left( {{{\left( {\rm{D}} \right)2} \over {({\rm{Dx}}2}}} \right.} \right) \cr & = {\left. {\left( {{1 \over {{\rm{DRI}}1}}} \right.} \right)^{}} + \left. {\left( {{1 \over {{\rm{DRI}}2}}} \right.} \right) \cr}$$

where D₁ and D₂ are the doses of drugs 1 and 2 taken together to achieve a specific level of effect. The doses of drug 1 and drug 2 alone resulting in the similar amount of action are Dx₁ and Dx₂. The dose-reduction indices for drugs 1 and 2 are DRI₁ and DRI₂, respectively.

### Apoptosis recognition with flow cytometry

The Annexin V-FITC apoptosis recognition kit (Miltenyi Biotec) was conducted to assess apoptosis. Sorafenib, hydroxychloroquine, and the synergistic combination dosage were conducted to cure MDA-MB-231 breast tumor cells for 48 h. Following centrifugation, the cells were resuspended in binding buffer, harvested using fluorescein isothiocyanate (FITC), and classified using Annexin V for 15 min at room temperature in the dark. Following two washes with 1×PBS, the cells were resuspended in binding buffer, propidium iodide was added, and they were harvested over 15 min at room temperature in dark [29]. The marked cells were examined via BD FACS flow cytometer (BD Biosciences).

### Investigation of the cell cycle inhibition with the flow cytometry

PI/RNA reagent (cell signaling) was used to do cell cycle analysis. The pellet was resuspended in 1 mL of PBS per 10^6 cells after the cells were centrifuged for five minutes at 1800 rpm to remove the supernatant. The particle was re-suspended for 60 min in 1 mL of cold ice 90% methanol following centrifugation and the removal of the supernatant. After centrifuging methanol-fixed cells for five minutes at room temperature at 1800 rpm, the cell pellet was resuspended in one PBS. The pellet was resuspended in 200 µL of cell cycle reagent and harvested at room temperature for 30 min after the cells were centrifuged once more at 1800 rpm over five minutes. Supernatant was discarded​ [30]. After being moved to a 5 mL flow tube, cell suspension models were prepared for Flowcytometry analysis. The stained cells were assessed using the BD FACS flow cytometer (BD Biosciences).

### Statistical analysis

Statistical analyses were performed using GraphPad Prism version 8.00 (GraphPad Software, San Diego, CA, USA). One-way ANOVA followed by Tukey’s multiple comparisons test (*p* < 0.05) was applied to apoptosis, and cell cycle data to compare multiple treatment groups. For combination index (CI) and dose reduction index (DRI) calculations, the Chou-Talalay method was employed using CompuSyn software (http://www.combosyn.com). All data are presented as mean ± standard deviation (SD).

**Fig. 1:**
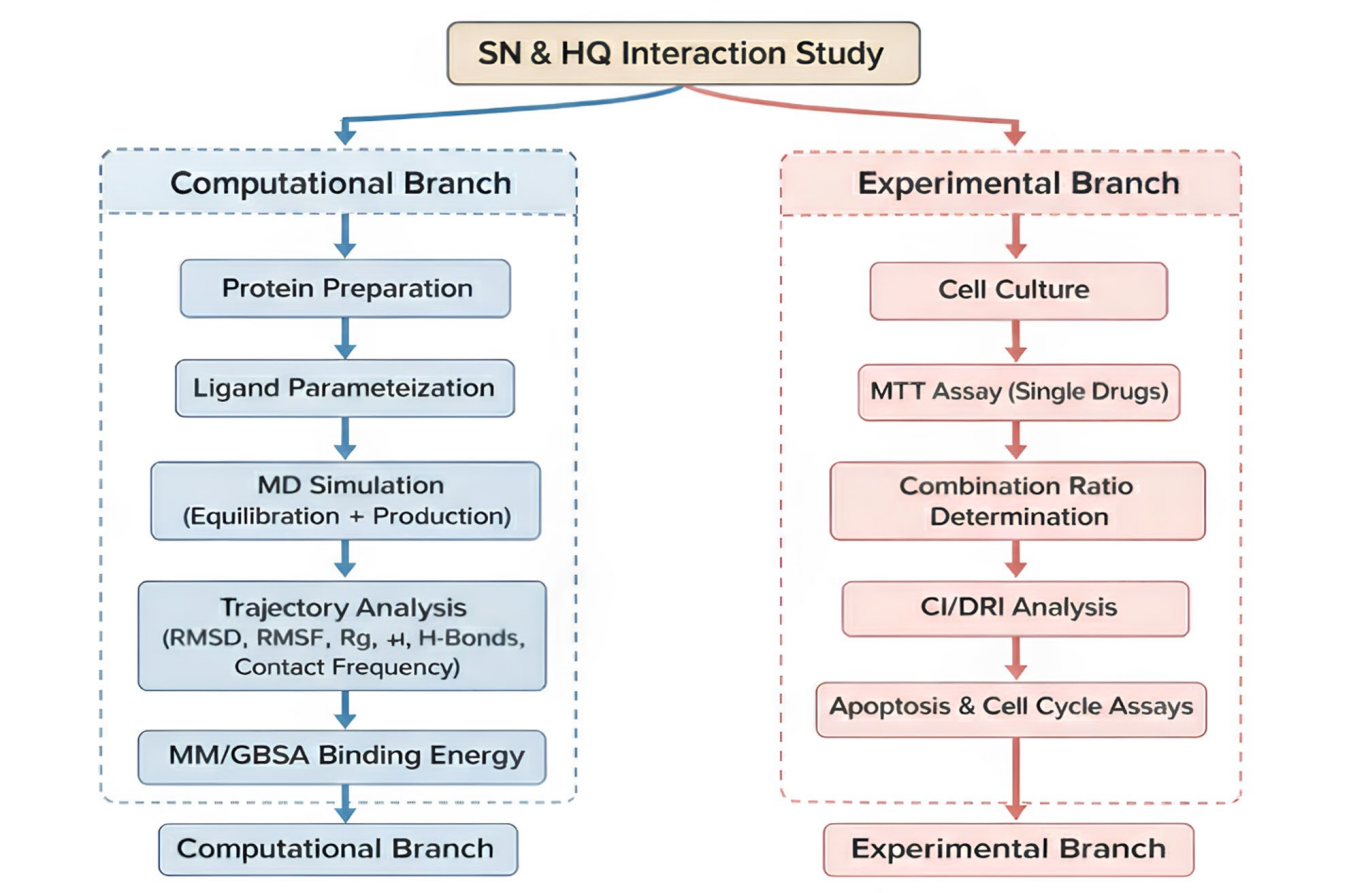
Schematic representation of the integrated computational and experimental workflow. The study design comprises two complementary branches. The computational branch (left) includes protein preparation, ligand parameterization, 100 ns MD simulation, trajectory analysis (RMSD, RMSF, Rg, H-bonds, contact frequency), and MM/GBSA binding free energy calculation. The experimental branch (right) comprises cell culture, MTT cytotoxicity assays for single drugs, combination ratio testing (SN: HQ = 1:1, 1:2, 2:1), CI/DRI analysis, and mechanistic validation via apoptosis and cell cycle assays. The integration of both approaches provides a comprehensive understanding of the synergistic mechanism

A schematic overview of the integrated computational and experimental workflow used in this study is provided in Fig. 1.

## Results

### Structural stability and dynamics for Raf–sorafenib complex

**Fig. 2:**
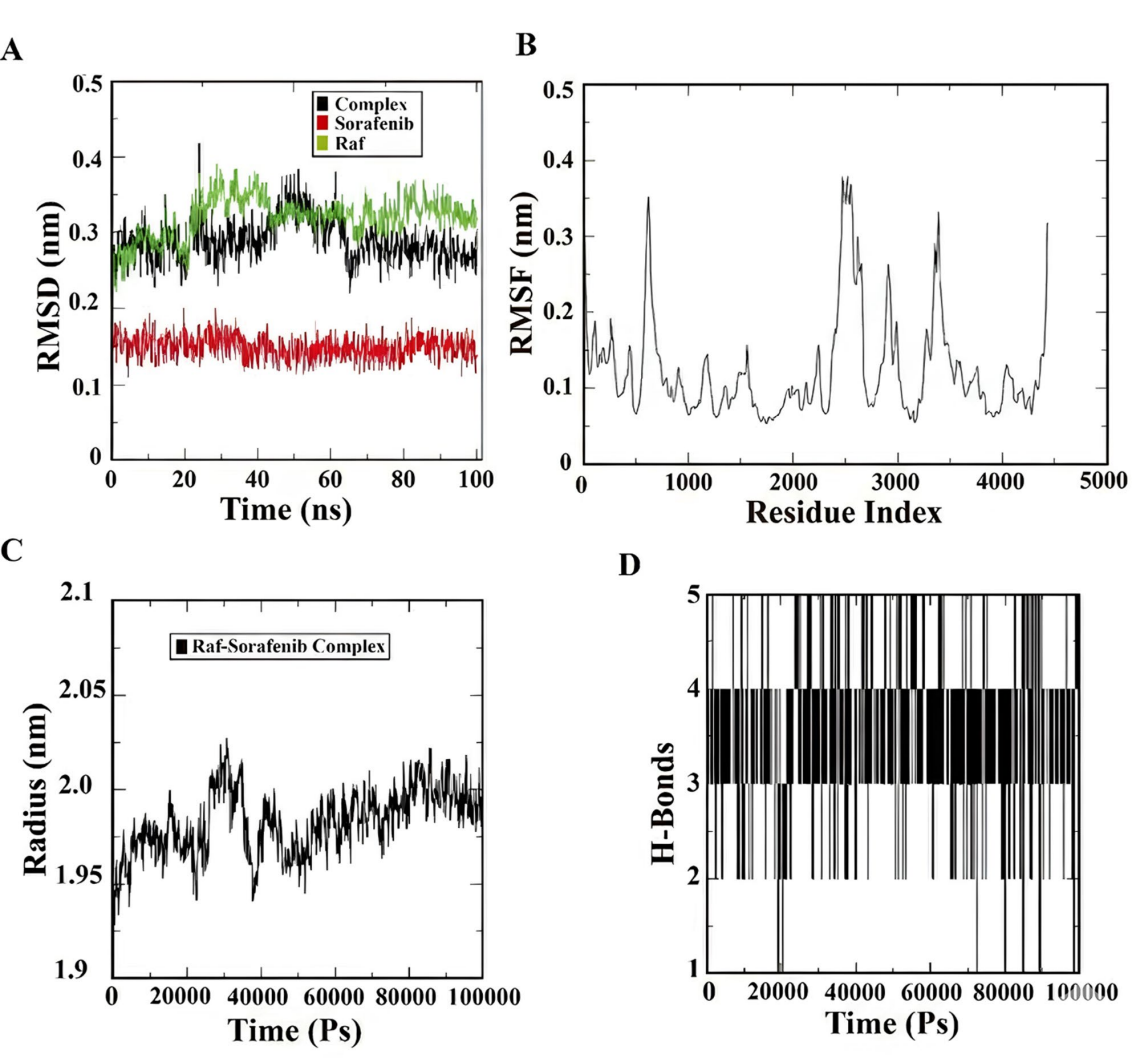
Molecular dynamics simulation analysis for Raf–Sorafenib complex. (**a**) the RMSD of ligand (Sorafenib) and protein backbone (Raf) across a 100 ns simulation period. (**b**) A plot of Cα atoms’ RMSF against residue index. (**c**) The complex’s gyration radius over time. (**d**) The amount of the intermolecular hydrogen bonds formed between Raf and Sorafenib during the course of the simulation

**Table 1:** Summary of key molecular dynamics parameters for the Raf-Sorafenib complex over the 100 ns simulation

Parameter	Mean ± SD
Protein backbone RMSD (nm)	0.24 ± 0.03
Ligand RMSD (nm)	0.15 ± 0.02
Radius of gyration (Rg, nm)	2.01 ± 0.02
Total solvent-accessible surface area (SASA, nm²)	195.2 ± 4.8
Number of intermolecular hydrogen bonds	3–4
Protein center of mass distance from box center (nm)	4.13 ± 0.02

We analyzed intermolecular hydrogen bonding patterns, root mean square fluctuation (RMSF), root mean square deviation (RMSD), and radius of gyration to evaluate the stability and conformational behavior of the Raf kinase in complex with sorafenib during 100 ns molecular dynamics simulations. The average values and standard deviations for these key MD parameters are summarized in Table 1, confirming the overall stability of the Raf-sorafenib complex throughout the trajectory.

### Root mean square deviation (RMSD) analysis

Over 100 ns, the root mean square deviation (RMSD) of ligand (Sorafenib) and protein Raf backbone in relation to the initial structure was tracked (Fig. 2a). After about 50 ns, the complex reached equilibrium, with the ligand RMSD stabilizing at about 0.15 nm and the protein RMSD stabilizing at around 0.25 nm. The low and convergent RMSD readings following the initial equilibration period show the complex’s general stability.

### Root mean square fluctuation (RMSF)

RMSF analysis of Cα atoms (Fig. 2b) revealed regions of flexibility within the Raf structure. The N- and C-terminal zones exhibited higher fluctuations (~ 0.3–0.5 nm), consistent with their solvent-exposed and unstructured nature. In contrast, the central catalytic domain (residues ~ 350–550) showed lower fluctuations (< 0.2 nm), indicating structural rigidity, likely due to ligand binding and stable secondary structure elements.

### Radius of gyration (Rg)

Through the simulation, the gyration radius for the whole complex and around its principal axes was constant at approximately 1.95–2.05 nm (Fig. 2c). The minimal variation in Rg suggests that the whole compactness and shape of the protein were maintained, including no significant unfolding or large-scale conformational variations during the simulation time.

### Intermolecular hydrogen bonds

Over time, quantity of hydrogen bonds between Raf and Sorafenib was monitored (Fig. 2d). A median of 3–4 stable hydrogen bonding was maintained, via occasional fluctuations between 2 and 5 bonds. The consistent presence of multiple hydrogen bonds indicates stable and specific interactions at the binding site, contributing to the complex’s stability.

### Stability of the Raf–sorafenib complex assessed by center of mass distance

**Fig. 3:**
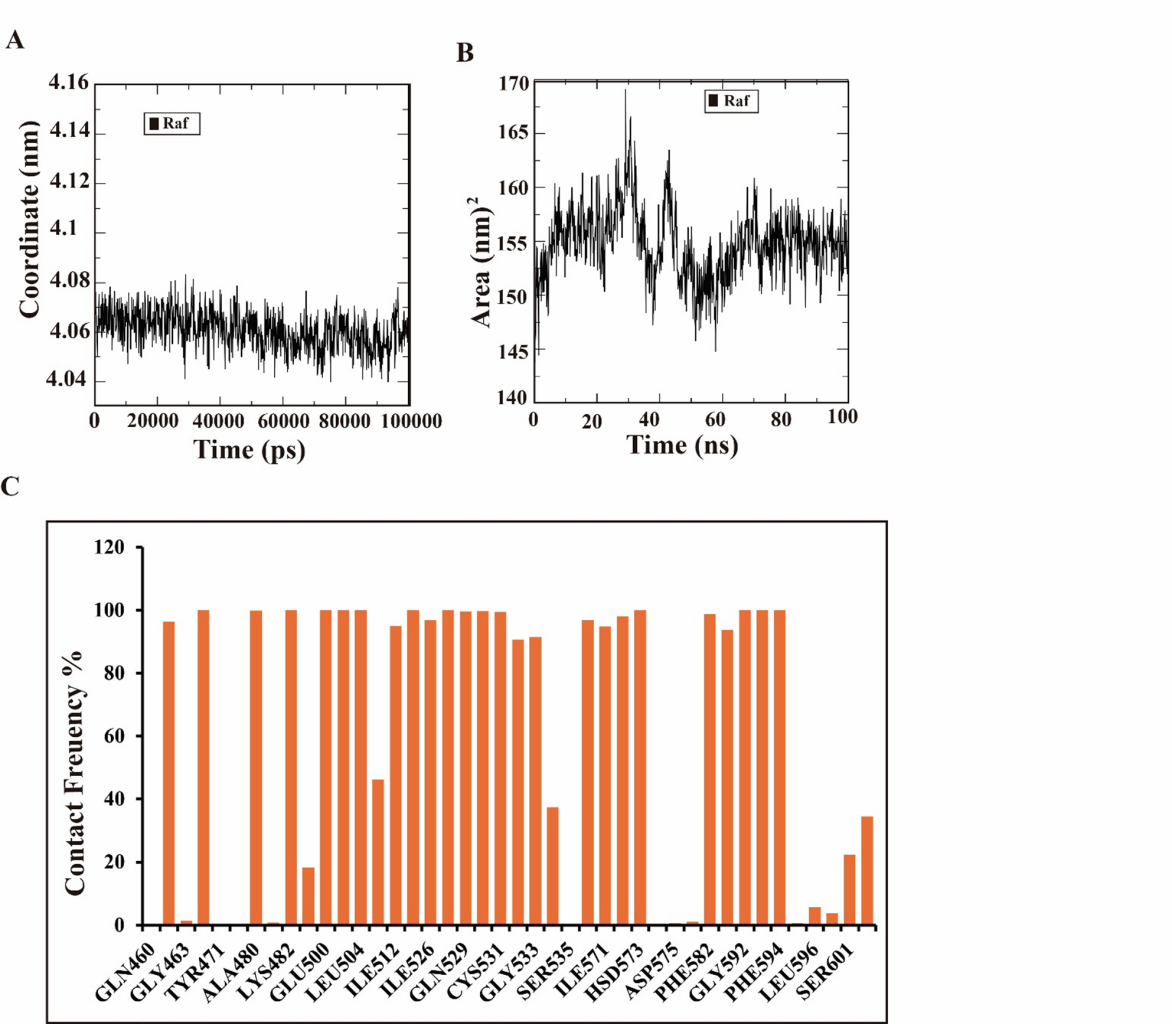
Molecular dynamics descriptors of the sorafenib–RAF complex: (**a**) center-of-mass distance, (**b**) total solvent-accessible surface area (SASA), and (**c**) contact frequency of significant RAF residues cooperating with sorafenib

**Table 2:** MM/GBSA binding free energy components for the Raf-Sorafenib complex calculated from the last 50 ns of the MD trajectory

Energy Component (kcal/mol)	Mean ± SD
Van der Waals (ΔVDWAALS)	-55.74 ± 3.06
Electrostatic (ΔEEL)	-42.98 ± 4.75
Total Gas-Phase Energy (ΔG_GAS)	-98.72 ± 5.05
Polar Solvation (ΔEGB)	+ 56.76 ± 3.69
Non-Polar Solvation (ΔESURF)	-7.94 ± 0.24
Total Solvation Energy (ΔG_SOLV)	+ 48.82 ± 3.65
Total Binding Free Energy (ΔG_bind)	-49.90 ± 2.69

To assess the overall stability of the simulation system, we tracked the center of mass (COM) distance of the Raf protein relative to the center of the simulation box over the 100 ns molecular dynamics trajectory. As shown in Fig. 3a, this distance remained consistently between 4.10 and 4.16 nm with minimal fluctuations throughout the simulation period. This stable COM distance indicates that the protein did not undergo significant drifting within the box, confirming that the system remained well-equilibrated and that the periodic boundary conditions were appropriately maintained. It is important to note that this measurement reflects protein positioning within the simulation box rather than direct protein-ligand binding proximity; the actual protein-ligand COM distance is substantially smaller (approximately 0.5–1.0 nm), consistent with stable binding within the active site. Detailed MM/GBSA binding free energy components for the Raf-sorafenib complex are presented in Table 3, revealing a total binding free energy of ΔG = -49.90 ± 2.69 kcal/mol. This value falls within the expected range (-40 to -60 kcal/mol) for high-affinity Type II kinase inhibitors, confirming stable complex formation [31, 32].

### Conformational stability of the Raf–sorafenib complex via solvent accessible surface area (SASA)

The Raf–Sorafenib complex’s SASA was examined to evaluate solvent exposure and conformational stability. Total SASA remained largely constant over the 100 ns trajectory, with only minor transient variations. This consistent SASA profile suggests that the complex secured a stablized folded state with no major structural rearrangements or unfolding, supporting the structural integrity of the bound complex (Fig. 3b).

### Contact frequency (CF)

Dynamic stability and binding interactions were assessed during the 100 ns molecular dynamics (MD) simulations, where the complex was fully equilibrated. Contact frequency (CF) analysis, through a cutoff distance of 4 Å, revealed that Sorafenib preserved persistent interactions among several key residues in the Raf binding pocket. The residues with the highest contact frequencies (> 80%) included VAL471, LEU513, HSD573, ILE591, GLY592, ASP593, PHE594, and GLU599, indicating their crucial role in stabilizing the inhibitor complex. Notably, residues such as ILE462, VAL470, LYS482, ALA480, ASN499, VAL503, GLU500, LEU504, THR507, ILE512, LEU513, ILE526, GLN529, THR528, TRP530, CYS531, GLU532, GLY533, SER534, LEU566, ILE571, ILE572, HSD573, ASN580, PHE582, LEU596, LYS600, SER601, and ARG602 also showed significant contact frequencies (> 40%), reflecting a broad and stable binding network (Fig. 3c). Trajectory visualization confirmed that Sorafenib remained deeply embedded in the hydrophobic cleft of Raf throughout the simulation, with no significant displacement or dissociation events.

### Sorafenib-protein complex binding free energy determined by MM/GBSA

The gmx_MMPBSA analysis using the Generalized Born (GB) implicit solvent model yielded a total binding free energy (ΔG_bind) of -49.90 ± 2.69 kcal/mol (mean ± SD) for the Sorafenib-Raf complex over 100 sampled frames. The decomposition of this energy reveals the driving forces behind binding. The interaction is strongly driven by favorable gas-phase contributions (ΔG_GAS = -98.72 ± 5.05 kcal/mol), which include favorable van der Waals (ΔVDWAALS = -55.74 ± 3.06 kcal/mol) and electrostatic (ΔEEL = -42.98 ± 4.75 kcal/mol) interactions. However, this favorable gas-phase energy is partially offset by an unfavorable polar solvation energy (ΔEGB = + 56.76 ± 3.69 kcal/mol), as the desolvation of polar groups upon binding incurs an energetic cost. In contrast, the non-polar solvation contribution (ΔESURF = -7.94 ± 0.24 kcal/mol), which corresponds to the burial of solvent-accessible surface area, is favorable and contributes to the net binding affinity. These results are consistent with the typical energetic profile of a high-affinity inhibitor binding to a hydrophobic kinase pocket [31].

### Effect of Sorafenib and hydroxychloroquine on cell viability

**Fig. 4:**
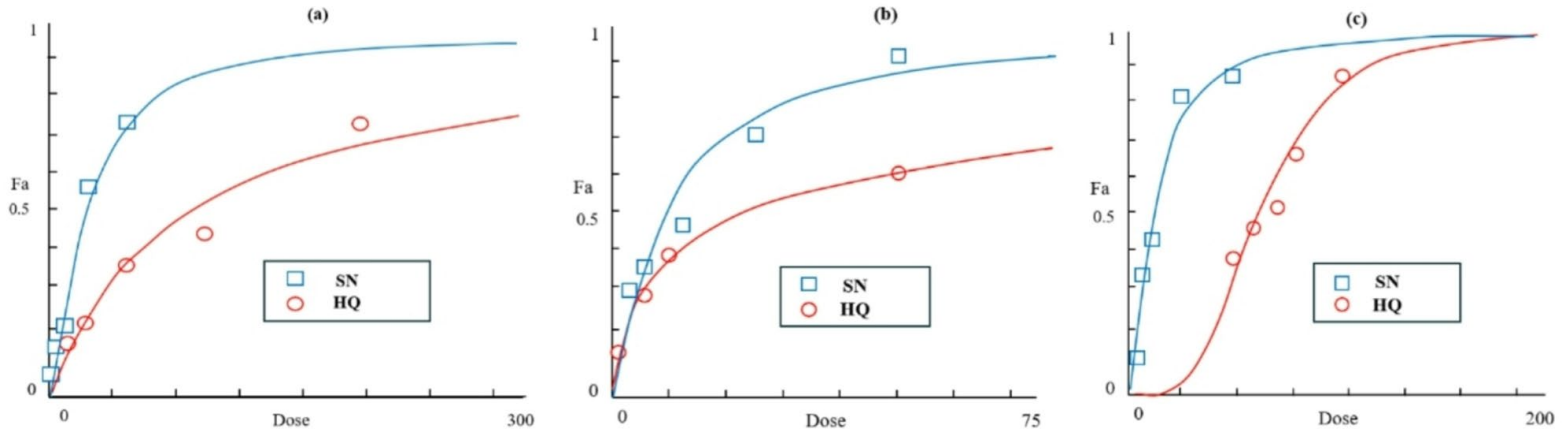
Dose–response MTT cytotoxicity assay of Sorafenib (SN) and Hydroxychloroquine (HQ) following 48 h treatment in (**a**) normal gingival fibroblasts, (**b**) MDA-MB-231 breast cancer cells, and (**c**) A549 lung cancer cells. Data were normalized to untreated controls (*n* = 5), and the fraction affected (Fa) was calculated as $Fa = {{\% \>{\rm{Cell}}\>{\rm{Viability}}} \over {100}}$. IC₅₀ values (µM) for SN were 23.1 (fibroblasts), 9.4 (MDA-MB-231), and 12 (A549), while HQ IC₅₀ values were 86.2, 23.6, and 62.4, respectively

The MTT method was used for assessing the cytotoxic effects of sorafenib and hydroxychloroquine (HQ) in lung A549 cancer cells, breast MDA-MB-231 cancer cells, and normal gingival fibroblasts. Increasing concentrations of each treatment were used to treat the cells; untreated cells in culture medium were used as controls. IC₅₀ values were calculated using CompuSyn software.

As shown in Fig. 4a, treatment of normal gingival fibroblasts with Sorafenib and HQ lead to dose-dependent decrease in cell viability, by cytotoxic effects detected at 23.1 µM and 86.2 µM, respectively (*n* = 5). Similarly, breast MDA-MB-231 tumor cells exhibited reduced viability upon drug treatment, with IC₅₀ values of 9.4 µM of Sorafenib and 23.6 µM of HQ (Fig. 3b, *n* = 5). In A549 lung cancer cells, both drugs also induced a dose-dependent decline in viability, with IC₅₀ values of 12 µM for Sorafenib and 62.4 µM for HQ (Fig. 4c).

Overall, both Sorafenib and HQ demonstrated a concentration-dependent inhibition of cell progression in all tested cell lines. Notably, IC₅₀ comparisons indicated that normal gingival fibroblasts were less sensitive to both drugs than cancerous cells, highlighting the selective cytotoxicity of these agents (Table 3).

**Table 3:** Sorafenib and Hydroxychloroquine IC₅₀ values of in different cell lines

Drug	IC₅₀		
	A549 (Lung cancer)	MDA-MB-231 (Breast cancer)	Normal gingival fibroblast
Sorafenib	12 µM	9.4 µM	23.1 µM
Hydroxychloroquine	62.4 µM	23.6 µM	86.2 µM

### Effect of sorafenib/hydroxychloroquine combination on breast MDA-MB-231 and lung A549 tumor cells

**Fig. 5:**
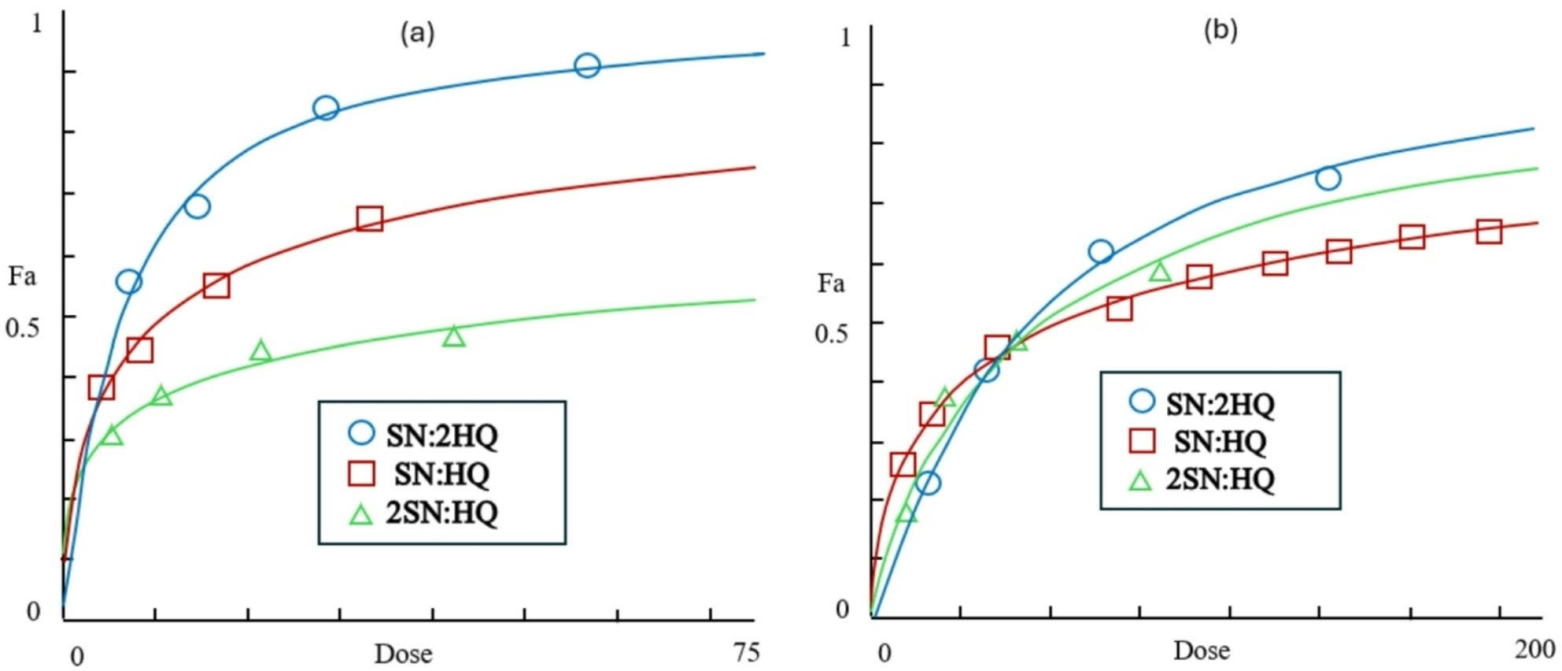
Dose–effect curves of Sorafenib (SN) and Hydroxychloroquine (HQ) combinations in (**a**) MDA-MB-231 and (**b**) A549 cells after 48 h treatment. Cytotoxicity was assessed by MTT assay, and the fraction affected (Fa) was calculated as $Fa = {{\% \>{\rm{Cell}}\>{\rm{Viability}}} \over {100}}$. Combination index (CI) analysis indicated strong synergy in MDA-MB-231 cells for SN:2HQ (CI = 0.32) and SN: HQ (CI = 0.66), whereas 2SN: HQ was antagonistic (CI = 3.7). In A549 cells, SN:2HQ was nearly additive (CI = 1.03), while SN: HQ (CI = 1.5) and 2SN: HQ (CI = 1.7) showed antagonism

Figure (5a) and (5b) illustrate the dose–effect curves for various combination ratios of Sorafenib (SN) and Hydroxychloroquine (HQ) on MDA-MB-231 breast cancer and A549 lung cancer cells, respectively, after 48 h of treatment. The fraction affected (Fa), representing the proportion of inhibited or dead cells, was calculated as $Fa = {{\% \>{\rm{Cell}}\>{\rm{Viability}}} \over {100}}$.

In both cell lines, *Fa* values increased progressively with increasing drug concentrations, demonstrating a clear dose-dependent cytotoxic response. However, the extent of inhibition varied according to the SN/HQ ratio.

For MDA-MB-231 cells (Fig. 5a), the SN:2HQ combination produced the highest *Fa* values across all concentrations, indicating the greatest cytotoxic activity and suggesting a strong synergistic effect when Hydroxychloroquine was in excess. The SN: HQ combination exhibited moderate inhibition, while 2SN: HQ showed the lowest *Fa* values, reflecting reduced efficacy when Sorafenib predominated.

A different dose-dependent pattern was observed in A549 cells (Fig. 5b). The SN:2HQ combination again achieved the highest cytotoxic effect, while SN: HQ ratio shows the least effect, and 2SN: HQ displayed a moderate response. This pattern indicates that while Sorafenib and Hydroxychloroquine act synergistically in both cell types, the strength of interaction is more prominent in breast MDA-MB-231 cancer cells.

**Fig. 6:**
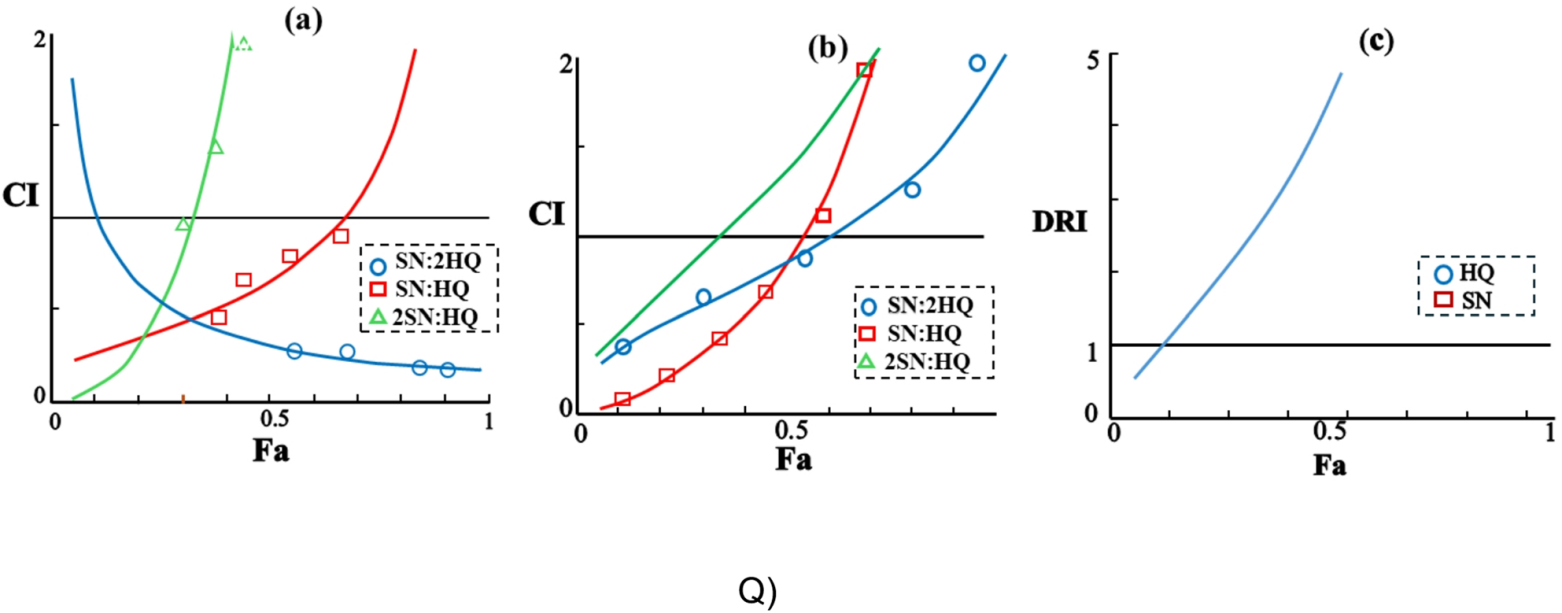
Combination index (CI) and dose reduction index (DRI) analyses of Sorafenib (SN) and Hydroxychloroquine (HQ) after 48 h treatment. (**a**) A549 and (**b**) MDA-MB-231 CI–Fa plots for fixed ratios (2SN:1HQ, 1SN:1HQ, 1SN:2HQ), where $Fa = {{\% \>{\rm{Cell}}\>{\rm{Viability}}} \over {100}}$. In MDA-MB-231 cells, 1SN:2HQ (CI = 0.32) and 1SN:1HQ (CI = 0.66) showed synergism, whereas 2SN:1HQ was antagonistic (CI = 3.78). All ratios were antagonistic in A549 cells (CI > 1). (**c**) DRI–Fa plot for the selective 2HQ:1SN ratio in MDA-MB-231 cells; at Fa = 0.5, DRI values were 4.68 (HQ) and 9.36 (SN), indicating marked dose-reduction potential

**Table 4:** Combination index (CI) values of various medication ratios in A549 lung tumor cells and MDA-MB-231 breast tumor cells

Combination Ratios	Combination Index (CI)	
	MDA-MB-231 Breast tumor cells	A549 lung tumor cells
SN:2HQ	0.32 (Highly synergistic)	1.03 (Nearly additive)
SN: HQ	0.66 (Synergistic)	1.5 (Antagonistic)
2SN: HQ	3.7 (Strongly antagonistic)	1.7 (Antagonistic)

Overall, these results the anticancer action of Sorafenib and Hydroxychloroquine combinations is ratio-dependent, with HQ-enriched doses (SN:2HQ) producing the most pronounced synergistic effect, particularly against MDA-MB-231 breast cancer cells.

The combination index (CI) analysis (Fig. 6a; Table 4) was conducted to evaluate the interaction between Sorafenib (SN) and Hydroxychloroquine (HQ) at different combination ratios (2SN:1HQ, 1SN:1HQ, and 1SN:2HQ) in breast MDA-MB-231 cancer cells. The analysis illustrated that the 1SN:1HQ and 1SN:2HQ ratios produced synergistic effects, with CI values of 0.66 and 0.32, respectively (CI < 1 indicates synergy). In contrast, the 2SN:1HQ ratio exhibited an antagonistic interaction, yielding a CI value of 3.78 (CI > 1 indicates antagonym).

As shown in Fig. 6b, the same combination ratios tested on A549 lung cancer cells displayed antagonistic interactions, suggesting that the synergistic potential observed in breast MDA-MB-231 cancer cells was absent in this cell line.

Towards assessing the potential for dose optimization, the DRI was determined and illustrated in Fig. 6c. The DRI plot demonstrates the degree to which each drug’s dose can be decreased when applied in combination to achieve a specific fractional effect (*Fa*). At *Fa = 0.5*, the DRI results were 4.68-fold for Hydroxychloroquine and 9.36-fold for Sorafenib, indicating their reduction effectiveness compared to single-drug.

**Table 5:** Dose reduction index (DRI) of Sorafenib and Hydroxychloroquine for the selective ratio (2HQ: SN) on breast cancer MDA-MB-231 at Fa = 0.5

Fa	Dose Hydroxychloroquine	Dose Sorafenib	DRI hydroxychloroquine	DRI Sorafenib
0.5	23.6	9.4	4.68	9.36

In summary, the combination of Sorafenib and Hydroxychloroquine exhibits a cell line-dependent effect, demonstrating antagonism in A549 lung cancer cells while showing synergistic cytotoxicity in MDA-MB-231 breast tumor cells for specific ratios. Moreover, the DRI for both drugs increase with higher fractional effects, reflecting greater dose reduction benefits as efficacy targets rise.

### Sorafenib/ hydroxychloroquine combination enhances killing in breast MDA-MB-231 cancer cells

**Fig. 7:**
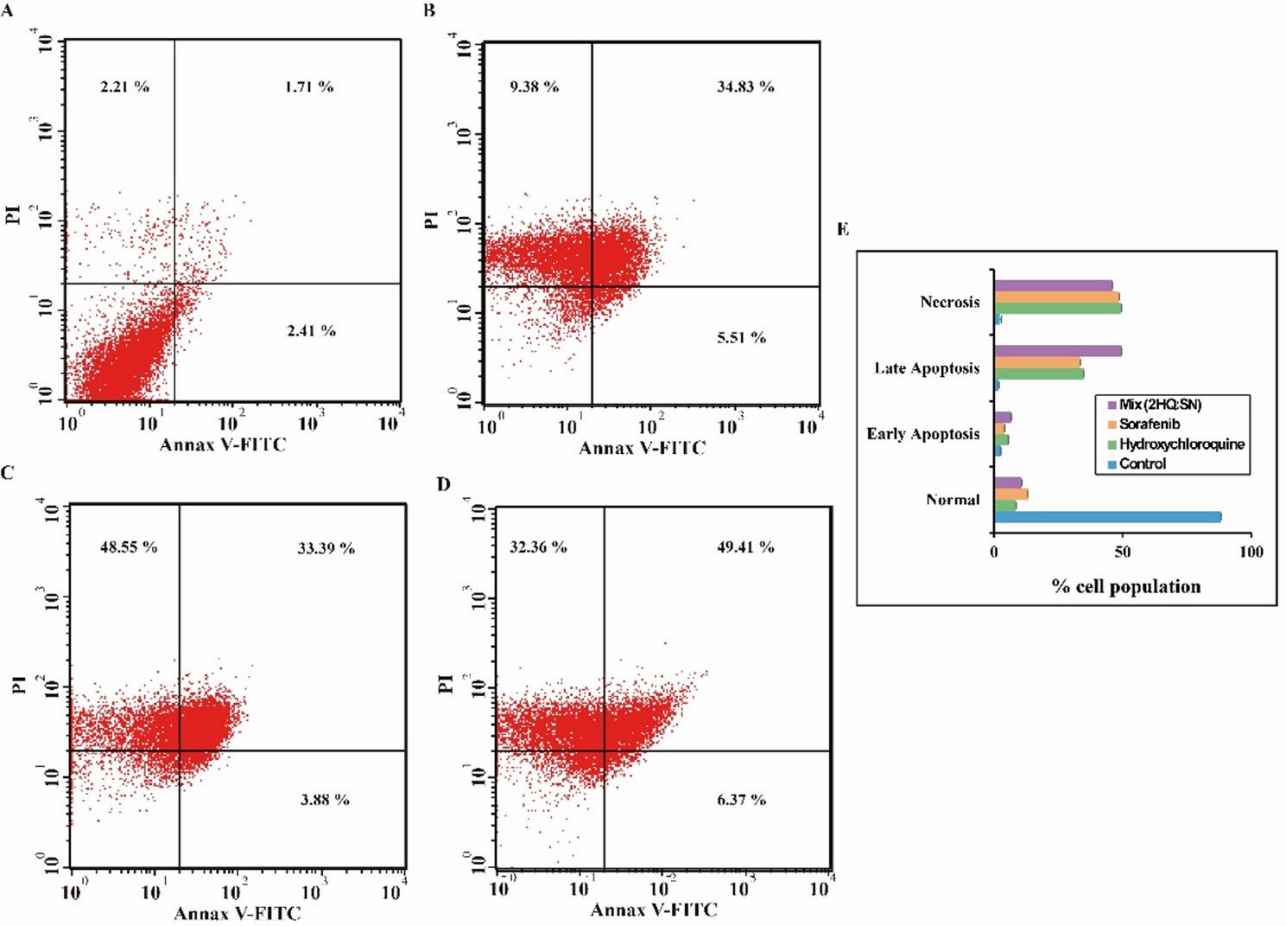
Apoptosis investigation via flow cytometry. Cells were stained with Annexin V-FITC and propidium iodide (PI) to distinguish viable (lower left), early apoptotic (lower right), late apoptotic (upper right), and necrotic (upper left) populations. Representative dot plots are shown for: (**a**) untreated control; (**b**) hydroxychloroquine (23.6 µM); (**c**) sorafenib (9.4 µM); and (**d**) their combination (5 µM HCQ + 1 µM Sorafenib). (**e**) Quantitative summary of cell populations across treatment groups. Hydroxychloroquine treatment significantly reduced viable cells (from 88.09% to 8.52%, ****p < 0.000001) and significantly increased early apoptosis (2.43% to 5.54%, ****p < 0.000001), late apoptosis (1.75% to 34.87%, ****p < 0.000001), and necrosis (2.23% to 49.40%, ****p < 0.000001) compared to control

As shown in Fig. 7, flow cytometry revealed that Sorafenib and Hydroxychloroquine individually promoted apoptosis for breast MDA-MB-231 cancer cells, via moderate early and late apoptotic populations. Sorafenib treated leaded to a 3.88% early and 33.39% late apoptotic/necrotic cells, while Hydroxychloroquine caused 5.51% early and 34.83% late apoptosis. Their combination with their dose reduction values significantly enhanced apoptosis (6.37% early and 49.41% late), compared to control cells that remained largely viable, confirming a synergistic apoptotic effect between the two drugs.

### Cell cycle distribution analysis of normal gingival fibroblast cells following drug treatment

**Fig. 8:**
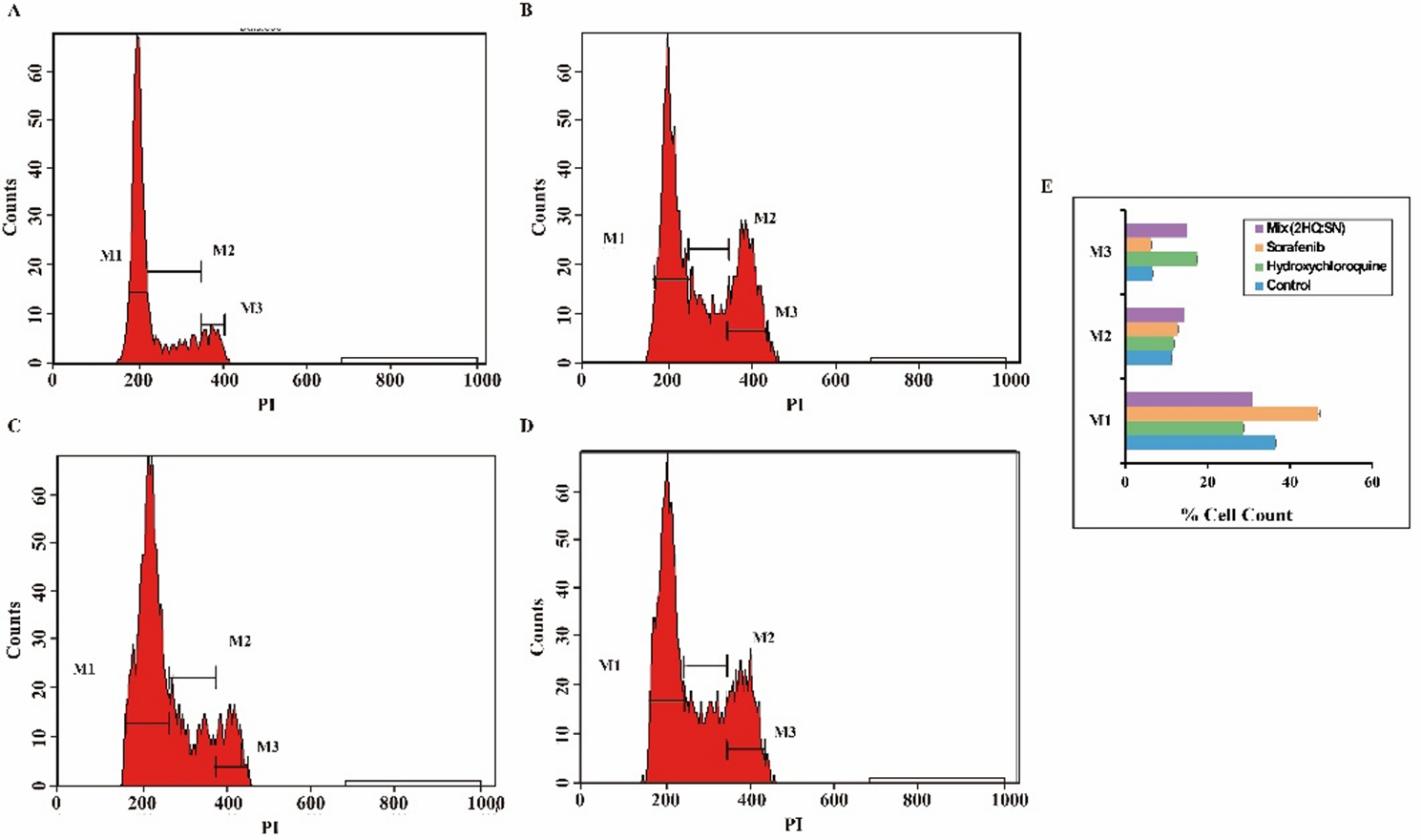
Flow Cytometry Investigation of cell cycle distribution in Normal Gingival Fibroblast cells. Cells were stained with PI to assess DNA content. Representative histograms show cell cycle distribution (G0/G1, M1; S, M2; G2/M, M3) for: (**a**) untreated control; (**b**) hydroxychloroquine (23.6 µM); (**c**) sorafenib (9.4 µM); and (**d**) combination treatment (5 µM HCQ + 1 µM Sorafenib). (**e**) Quantitative bar graph of phase distribution across groups. Two-way ANOVA demonstrated significant treatment effects (****P < 0.0001), phase effects (*P* < 0.0001), and interaction (*P* < 0.0001), indicating differential cell cycle alterations by treatments. Data are mean ± SD (*n* = 3)

As shown in Fig. 8, flow cytometry analysis examined the effects of hydroxychloroquine (HQ), sorafenib, and their combination on the cell cycle of normal gingival fibroblasts using propidium iodide staining. Control cells predominantly resided in the G0/G1 phase (63.83%), with 21.31% in S and 11.38% in G2/M. HQ treatment reduced the G0/G1 population to 49.52% and increased G2/M to 30.00%, while sorafenib caused only minor changes (61.42% G0/G1, 22.82% S, 14.74% G2/M). The combination treatment with its dose reduction produced a pattern similar to HQ alone (51.53% G0/G1, 20.00% S, 26.54% G2/M). These results suggest that the tested doses, though cytotoxic to cancer cells, do not significantly disrupt the cell cycle of normal fibroblasts.

## Discussion

This study elucidates the structural dynamics, cytotoxic efficacy, and synergistic potential of Sorafenib, alone and in combination with Hydroxychloroquine, against cancer cell lines. Our integrated approach, combining in silico molecular dynamics (MD) simulations with in vitro biological assays, provides a multi-faceted understanding of the compound’s mechanism of action and its enhanced effect when paired with an autophagy inhibitor.

The MD simulations confirm that Sorafenib forms a highly stable complex with Raf kinase, a key target in its anti-proliferative mechanism. The rapid equilibration and low RMSD values of both protein (0.24 ± 0.03 nm) and ligand (0.15 ± 0.02 nm) throughout the 100 ns simulation (Fig. 2a; Table 1) are indicative of a rigid binding mode. This structural stability is consistent with Sorafenib’s role as a Type II kinase inhibitor, which preferentially binds to and stabilizes the inactive DFG-out conformation of the kinase [33]. In this conformation, the conserved DFG motif (Asp-Phe-Gly) at the activation loop flips outward, creating a hydrophobic pocket adjacent to the ATP-binding site that accommodates Type II inhibitors.

The biological significance of this binding mode is profound. By locking Raf in its inactive conformation, Sorafenib prevents its phosphorylation and subsequent activation of the MAPK/ERK signaling cascade (MEK → ERK). This pathway is constitutively activated in KRAS-mutant cancers, driving uncontrolled proliferation and survival. Thus, the stable complex observed in our simulations provides a structural rationale for Sorafenib’s ability to intercept oncogenic signaling at its source.

The low RMSF values observed within the catalytic domain (residues 350–550), particularly in regions corresponding to the DFG motif and the catalytic loop, underscore this ligand-induced stabilization. In contrast, the higher fluctuations at the N- and C-terminal regions (~ 0.3–0.5 nm) are consistent with their solvent-exposed, flexible nature and are unlikely to affect inhibitor binding. The maintenance of a stable radius of gyration (2.01 ± 0.02 nm) and SASA (195.2 ± 4.8 nm²) throughout the simulation further confirms that the complex maintains its structural integrity without undergoing major conformational rearrangements or unfolding (Figs. 2c and 3b).

Critically, the interaction network revealed by contact frequency analysis aligns with known pharmacophore features for Raf inhibition. The persistent contacts with residues in the DFG motif (ASP593, PHE594), the catalytic spine (LEU513, ILE572, HSD573), and the C-helix region (GLU501, often numbered as 500 in other isoforms) are hallmarks of Type II binding [34, 35]. The MM/GBSA-derived binding free energy of -49.90 ± 2.69 kcal/mol (Table 2) quantitatively confirms the high affinity of Sorafenib for Raf kinase. This value is within the range expected for high-affinity Type II inhibitors targeting the DFG-out conformation, which typically exhibit binding energies between − 40 and − 60 kcal/mol for stable complexes [31, 32]. Decomposition of the binding energy reveals the thermodynamic forces driving this interaction. The binding is overwhelmingly driven by favorable gas-phase contributions (-98.72 ± 5.05 kcal/mol), which include both van der Waals (-55.74 ± 3.06 kcal/mol) and electrostatic (-42.98 ± 4.75 kcal/mol) interactions. The substantial van der Waals component reflects the snug fit of Sorafenib within the hydrophobic binding cleft, while the electrostatic contribution indicates specific polar interactions with key residues.

However, this favorable gas-phase energy is partially offset by an unfavorable polar solvation energy (+ 56.76 ± 3.69 kcal/mol), representing the energetic cost of desolvating both the ligand and the binding pocket upon complex formation. This desolvation penalty is typical for buried binding sites and is compensated by the formation of specific protein-ligand interactions. The favorable non-polar solvation term (-7.94 ± 0.24 kcal/mol) corresponds to the burial of hydrophobic surface area, further stabilizing the complex.

This energetic profile—strong gas-phase interactions balanced against a significant desolvation penalty—is characteristic of high-affinity inhibitors and explains the potent anti-proliferative effects observed in our cellular assays (IC50 values of 9.4 µM in MDA-MB-231 and 12 µM in A549 cells). The correlation between binding affinity and cellular potency supports the notion that Raf inhibition is a primary mechanism of Sorafenib’s cytotoxic activity.

The contact frequency analysis (Fig. 3c) identified several residues that play critical roles in stabilizing the Raf-Sorafenib complex. Residues with the highest contact frequencies (> 80%) included VAL471, LEU513, HSD573, ILE591, ASP593, PHE594, and GLU599. These residues form the core of the binding pocket and are directly involved in inhibitor recognition and stabilization.

Particularly noteworthy are the interactions with residues of the DFG motif (ASP593, PHE594), which are hallmark features of Type II inhibition. The DFG-out conformation positions these residues to create the hydrophobic pocket that accommodates the inhibitor. The persistent hydrogen bonding (average 3–4 bonds) (Fig. 2c) with residues such as ASP593 and GLU599 provides additional stabilization and contributes to the specificity of binding.

Understanding these residue-specific interactions has important implications for predicting and interpreting drug resistance mutations. In clinical settings, resistance to kinase inhibitors often arises through mutations that disrupt critical protein-ligand contacts or destabilize the inactive kinase conformation. For example, mutations at residues corresponding to VAL471 or LEU513 (analogous to the “gatekeeper” residue in other kinases) could sterically hinder inhibitor binding or alter pocket geometry. Similarly, mutations within the DFG motif (e.g., ASP593→Val or Phe) could prevent the conformational transition required for Type II inhibitor binding. By mapping these interactions, our study provides a structural framework for understanding potential resistance mechanisms that may emerge with Sorafenib therapy and could guide the development of next-generation inhibitors.

Cytotoxic Profile and Selective Synergy with Hydroxychloroquine. The MTT assays demonstrated the expected cytotoxic profile of Sorafenib, with IC₅₀ results in the low micromolar scale for MDA-MB-231 (9.4 µM) and A549 (12 µM) cells, consistent with its known activity against various carcinomas [9]. Notably, normal gingival fibroblasts exhibited higher IC₅₀ values (23.1 µM), suggesting a degree of selective toxicity towards cancer cells, a finding aligned with the therapeutic window observed clinically, albeit in a different cellular context (Fig. 3; Table 3) [36]. Hydroxychloroquine (HQ), an autophagy inhibitor, also showed cytotoxicity, with tumor cells being more sensitive than normal cells.

The most clinically significant finding of this study is the striking cell line-dependent synergy observed between Sorafenib and Hydroxychloroquine. While the combination exhibited strong synergy in MDA-MB-231 breast cancer cells (CI = 0.32 for SN:2HQ ratio), it showed antagonism in A549 lung cancer cells across all tested ratios (Fig. 6a; Table 4). This dichotomy underscores the context-specific nature of drug interactions and highlights the need for biomarker-driven patient stratification in clinical applications. This synergy is biologically validated by the flow cytometry data, where the combination at reduced doses (5 µM HQ + 1 µM SN) induced a substantial increase in late apoptosis (49.41%) compared to either agent alone (Fig. 7). This enhancement aligns with the proposed mechanism wherein Sorafenib, by inhibiting the MAPK pathway, induces stress and protective autophagy; HQ concurrently blocks autophagic flux, leading to apoptotic catastrophe [37, 38]. Despite the cell line-dependent effects, the synergy observed in MDA-MB-231 cells carries significant translational potential. The high Dose Reduction Indices (DRI) for both drugs at Fa = 0.5 (9.36-fold for Sorafenib and 4.68-fold for Hydroxychloroquine; Table 5) indicate that effective cytotoxicity can be achieved at substantially lower doses than those required for single-agent activity. This dose reduction has important clinical implications:

Sorafenib is associated with dose-limiting toxicities including hand-foot skin reaction, diarrhea, and fatigue. Hydroxychloroquine, while generally well-tolerated, can cause retinopathy with prolonged high-dose use. The ability to achieve therapeutic efficacy at lower doses could mitigate these adverse effects, improving patient quality of life and treatment adherence.

By targeting two complementary pathways (MAPK/ERK signaling and autophagy), the combination may delay or prevent the emergence of resistance mechanisms that frequently limit the durability of single-agent targeted therapies.

The favorable safety profile of the combination on normal cells (Fig. 8), with minimal disruption to the cell cycle of gingival fibroblasts at synergistic doses, suggests a degree of cancer-selective cytotoxicity. This selectivity likely stems from the heightened stress and autophagy dependence of cancer cells compared to their normal counterparts.

Conversely, the antagonistic interaction observed in A549 lung cancer cells across all ratios (Table 4) reveals a critical cell line-dependent response. This dichotomy may be explained by differential reliance on autophagy for survival, variations in upstream signaling pathways that modulate the efficacy of Raf inhibition, or distinct baseline metabolic states [39]. This finding cautions against the blanket application of this combination and emphasizes the need for biomarker-driven patient stratification.

Therapeutic implications and little effects on the normal cell cycle. Fortunately, normal gingival fibroblasts’ cell cycle was not notably disrupted by the combined treatment at dosages that were synergistic for MDA-MB-231 breast tumor cells (Fig. 8). While HQ alone induced a G2/M accumulation phenomenon previously linked to autophagy inhibition and mitotic disruption [40] the combination did not exacerbate this effect. This suggests that the synergistic apoptotic trigger may be more specific to the dysregulated signaling and stress response pathways inherent to cancer cells, sparing normal cells from profound cell cycle arrest at these doses.

## Conclusion

In conclusion, our integrated computational-experimental approach demonstrates that Sorafenib forms a stable, high-affinity complex with Raf kinase through a Type II binding mode involving critical interactions with DFG motif residues and surrounding hydrophobic pockets. The energetic drivers of binding—favorable gas-phase interactions partially offset by a desolvation penalty—are characteristic of potent kinase inhibitors and correlate with the observed anti-proliferative activity. The combination of Sorafenib with Hydroxychloroquine yields a potent, ratio-dependent synergy in MDA-MB-231 breast cancer cells, mechanistically driven by enhanced apoptosis, while exhibiting antagonism in A549 lung cancer cells, highlighting the critical importance of tumor context. The favorable dose reduction indices and limited impact on normal cell cycle progression support the further investigation of this combination as a targeted strategy to enhance Sorafenib’s efficacy and potentially overcome resistance, particularly in breast cancer models where autophagy serves as a critical survival pathway.

## Data Availability

The datasets generated and/or analyzed during the current study are available from the corresponding author on reasonable request.

## Abbreviations

KRAS: Kirsten rat sarcoma viral oncogene homolog
Raf: Rapidly accelerated fibrosarcoma kinase
MAPK/ERK: Mitogen-activated protein kinase/extracellular signal-regulated kinase
PI3K/AKT: Phosphatidylinositol 3-kinase/protein kinase B
HQ: Hydroxychloroquine
SN: Sorafenib
MD: Molecular dynamics
RMSD: Root mean square deviation
RMSF: Root mean square fluctuation
Rg: Radius of gyration
SASA: Solvent-accessible surface area
MM/GBSA: Molecular mechanics/generalized Born surface area
IC50: Half maximal inhibitory concentration
Fa: Fraction affected
CI: Combination index
DRI: Dose reduction index
MTT: 3-(4,5-Dimethylthiazol-2-yl)-2,5-diphenyltetrazolium bromide
PI: Propidium iodide
FITC: Fluorescein isothiocyanate
ANOVA: Analysis of variance​
PBC: Periodic boundary conditions
NVT/NPT: Constant volume/temperature and constant pressure/temperature ensembles
PME: Particle mesh Ewald
CF: Contact frequency
G_bind: Binding free energy​
FBS: Fetal bovine serum
G0/G1, S, G2/M: Cell cycle phases
CERRMA: Center of Excellence for Research in Regenerative Medicine and its Application
